# The Voice of Families: Perceptions of Family-Centred Practices and Natural Environments in Early Intervention in Spain

**DOI:** 10.3390/children12081068

**Published:** 2025-08-14

**Authors:** Mónica Montaño-Merchán, Roberto Sanz-Ponce, Laura Padilla-Bautista, Joana Calero-Plaza

**Affiliations:** Faculty of Teaching and Educational Sciences, Catholic University of Valencia, 46110 Valencia, Spain; monica.montano@ucv.es (M.M.-M.); laura.padilla@ucv.es (L.P.-B.); joana.calero@ucv.es (J.C.-P.)

**Keywords:** early intervention, family-centred practices, routine-based model, natural environments, families’ perceptions, transdisciplinary intervention, family participation

## Abstract

**Highlights:**

**What are the main findings?**

- Coexistence of the traditional clinical model and the model based on routines and natural environments.
- Families’ request for more personalized models, where decision-making responsibility is shared.

**What is the implication of the main finding?**

- The need to modify the intervention model and gradually introduce models based on routines and natural environments.
- The need to involve families in the support and decision-making process.

**Abstract:**

The family-centred Early Intervention model based on routines and natural environments has been widely supported by international evidence in recent decades. Within this framework, Family-Centred Practices (FCP) and their development in natural environments have emerged as an evidence-based intervention model of reference, promoting parental empowerment, shared decision-making, and functional intervention through daily routines. However, its effective implementation in real contexts presents multiple challenges, especially from the perspective of families receiving the service. **Background/Objectives:** This study explores the experiences, meanings, and assessments of Spanish families with children who have disabilities or developmental difficulties in relation to the application of these professional practices. This study is carried out in the Spanish context, since Campus Capacitas (Campus Capacitas—Catholic University of Valencia, Spain) has been implementing, in recent years, the family-centred model as a model of early intervention. **Methods:** A qualitative, descriptive, and interpretative methodology was used. Data collection was carried out through semi-structured interviews and discussion groups with 30 families from the 17 Spanish autonomous communities. Data analysis was carried out through thematic coding following criteria of qualitative rigour such as triangulation and theoretical saturation. **Results:** The findings show a significant gap between the theoretical model of family-centred practices and their practical application. Families who have experienced a clinical model criticise the absence of personalised intervention, unidirectional communication, as well as lack of participation in decision-making. In that sense, it is the different specialists of the early intervention team who are responsible for making intervention decisions. Therefore, these families demand more emotional and educational support. On the other hand, other families report positive experiences associated with collaborative, transdisciplinary, and home-based models based on a family-centred model. **Conclusions:** The results highlight the urgent need to move towards early intervention that strengthens the active role of families, promotes professional co-responsibility, and adapts to real child development environments, in line with international recommendations. Regarding future lines of research, we are committed to the development of longitudinal studies on the sustainable effects of interventions centred on families and on the global development of children and families. To carry out comparative studies between autonomous communities, to assess the influence of regulatory factors and regional resources on the practices implemented, as well as to carry out triangulation studies of the professional practices implemented, incorporating the perspectives of professionals and other intervention agents to enrich the analysis.

## 1. Introduction

In recent decades, the Early Intervention (EI) paradigm has undergone a substantial transformation, shifting from clinical models focused exclusively on the child to more systemic, ecological, and collaborative approaches, where the family is recognised as an active and essential agent in the child development intervention process [1,2]. Within this framework, Family-Centred Practices (FCP) and their development in natural environments have emerged as an evidence-based intervention model of reference, promoting parental empowerment, shared decision-making, and functional intervention through daily routines [2,3].

The implementation of these practices responds to a broad conception of child development that recognises the decisive influence of the family and community environment [4]. In this vein, it has been pointed out that inclusive pedagogical practices are strongly associated with quality in early childhood education, highlighting the fundamental role of family involvement in educational processes from early childhood [5]. In this context, Robin McWilliam’s Routines-Based Model has been one of the most internationally influential and has been adopted in different sociocultural contexts [6,7]. This model can be implemented in any family, regardless of its sociocultural context. This model proposes that intervention be based on the needs identified by the families themselves and that actions be carried out in the child’s everyday contexts, such as the home, school, or community. In this sense, it promotes individualised planning of functional objectives based on daily routines, as well as professional support aimed at empowering and actively involving primary caregivers.

Authors such as Dunst et al. [3] and Subiñas et al. [8] have emphasised that the quality of the intervention is closely related to the level of parental empowerment and the perception of family competence. In this sense, it is the family-centred model that adapts to different types of families or cultures, and not the family or the culture that must adapt to the model. Empirical evidence shows that practices based on routines and developed in natural environments improve child participation, strengthen family bonds, and facilitate functional learning [8,9]. This intervention model has been validated in various contexts, including its recent application in Spain and Latin America, using tools such as the Routine-Based Interview (RBI), ecomaps, and fidelity rubrics, which enable systematic family-centred planning [10]. In this regard, studies conducted in these settings have shown significant benefits in family quality of life, perceptions of parental competence, and children’s functional participation in everyday contexts [8,11]. This evidence supports a progressive evolution in the incorporation of these practices, although a notable gap persists between the theoretical model and its actual implementation in Child Development and Early Intervention Centres (CDIAT) [12].

The literature indicates that many professionals still operate under an expert model, where decisions are made without the active and effective participation of the family, which limits the effectiveness of interventions and generates frustration among caregivers [4,13]. Research highlights the benefits of interventions focused on the home environment compared to those developed exclusively in clinical contexts, pointing to improvements in children’s achievements and the overall well-being of families [14,15]. In the case of Spain, recent research warns of the presence of interventions that are still focused on the clinical model, with little family involvement, insufficient institutional flexibility, and low training in the intervention model proposed by McWilliam [2,11,15].

The results of the aforementioned research [2,11,15] reveal that most studies conducted on PCF in Spain focus on the perceptions of professionals and that there is still limited scientific output that directly reflects the voices of families in relation to the implementation of professional practices [16]. This knowledge gap limits our understanding of how theoretical practices are experienced in everyday family life, as well as their barriers, contradictions, and potential.

In this sense, this study responds to this need by exploring the perceptions and experiences of Spanish families with children who have disabilities or developmental difficulties in relation to the professional early intervention practices they receive to determine which ones are centred on the routine-based model and developed in natural environments. The main purpose of this work is to analyse, through a qualitative methodology, the degree of actual implementation of PCF in the Spanish context, from the perspective of the families themselves, identifying points of disconnection between the theoretical model and its practical application, as well as possible areas for improvement and professional adjustment. Ultimately, this analysis seeks to contribute to improving the quality and consistency of AT services, promoting more humanised, contextualised and effective practices. In order to do so, two key research questions are posed: Has the family-centred model in early intervention been widely implemented in Spain? And, if so, what benefits does it provide according to the opinions of the families?

### Brief Literature Review

Early Intervention is currently undergoing an evolution from a clinical model, centred on the identification and treatment of deficits in the child, to a social and systemic approach, based on the interaction between the child, his family, and the environments in which he develops. This paradigm shift is based on the Ecological Theory of Human Development [17], which conceives the family as an open system made up of interdependent subsystems. In this framework, intervention is understood as an integrative and global process, which aims not only to improve the competencies of the child with difficulties but also to strengthen his or her immediate context, favouring his or her autonomy, social integration, and optimal development [18,19].

The change lies in the progressive centrality of the family and natural environments in the intervention, shifting the exclusive focus from the child to joint work with families as active and co-responsible agents in the intervention process. Evidence indicates that family-centred practices (FCP) promote parental self-competence and empowerment, improve family quality of life, and optimise the use of resources and daily routines as learning opportunities for the child and family [20,21]. This approach respects the family’s priorities, expectations, and needs, strengthening their capacity to make informed decisions and actively participate in the planning and implementation of interventions [22,23].

In this context, the Model Based on Routines and Natural Environments proposed by McWilliam [6] represents a methodological concretization of the ecological perspective, integrating support actions in the daily activities of the child and family. This model proposes that interventions should be carried out in significant environments, such as the home, community spaces, and the school, to facilitate the generalisation of learning and guarantee its functionality [24,25]. The child’s participation in these environments, in addition to promoting their autonomy, expands opportunities for interaction and learning, reinforcing their social participation and reducing barriers to their inclusion [26,27]. There are also other models that combine the work of professionals with the support of families, such as, for example, the Supporting Play, Exploration, and Early Development Intervention (SPEEDI) [28], for premature infants; the Coping with and Caring for Infants with Special Needs (COPCA) [29], which implements an active role of the family together with the coaching work of the physical therapist; and the Goals–Activity–Motor Enrichment (GAME) [30], which has obtained promising results with infants at high risk for cerebral palsy.

Together with the family context, the school context also becomes relevant as one of the environments in which the child spends a large part of his or her time, and its involvement is key to ensure the coherence of common objectives and strategies [31]. Therefore, it is essential to establish a collaborative relationship with the educational staff of the school. In this sense, collaboration between TA professionals, family and educational staff is vital, as it enables more consistent interventions, aligned with the real needs of the infant and respectful of his or her learning pace [32].

## 2. Materials and Methods

This study is part of a qualitative, exploratory-descriptive approach with an interpretative phenomenological orientation. The aim was to gain an in-depth understanding of the experiences, perceptions, and meanings that Spanish families attribute to the professional practices they receive in the field of Early Intervention (EI), with a special focus on the Family-Centred Model based on Routines and Natural Environments [33,34].

The sample consists of 30 families with children with disabilities or developmental risks (mothers, fathers, or both parents) from the 17 Spanish autonomous communities, referred by paediatric specialists by medical report (Table 1). In addition, Child Development and Early Intervention Centres (CDIAT) conduct an assessment to understand the needs of each child and their families and plan the most appropriate intervention. Participation was voluntary and diverse in terms of age, sociodemographic profile, and experience in Child Development and Early Intervention Centres (CDIAT). Recruitment was carried out through social networks, parent groups, and within the framework of the First International Meeting on Early Intervention Research and Updates (Campus Capacitas—Catholic University of Valencia, Spain). The sample selection was non-random, and any families who wished to participate were welcome, provided they were already in Child Development and Early Intervention Centres (CDIAT) for at least one year and, therefore, had a paediatric report. The study was conducted in accordance with the Declaration of Helsinki and approved by the Institutional Review Board. Approval was obtained from the Ethics Committee of the Catholic University of Valencia (protocol code CEI/UCV/2018-2019/111), approved on 20 January 2020.

In-depth semi-structured interviews were used as a data collection tool. Interviews were conducted via video call (Skype, 18; Facetime, 4) or in person (8). All interviews were recorded. Families were previously informed of the research objective, requested a series of demographic data, and signed an informed consent form. The interviews were subsequently fully transcribed. The interviews followed the same structure: introduction, request for some sociodemographic data, and a series of open-ended questions aimed at exploring in depth the families’ experiences regarding the professional practices received, the type of intervention applied, and their active or peripheral participation in the process. The interview consists of 32 questions. Sample questions include: Could you explain what the assessment process was like (location, who was present, how it was done, and collection of supports and needs of the child and family)?; are you asked about the needs and priorities of your child and family within your daily routines?; how were the goals of the intervention established?; or has an agreement been established with you (and perhaps with other family members who are with your child) regarding the intervention and its development?

**Figure 1:**
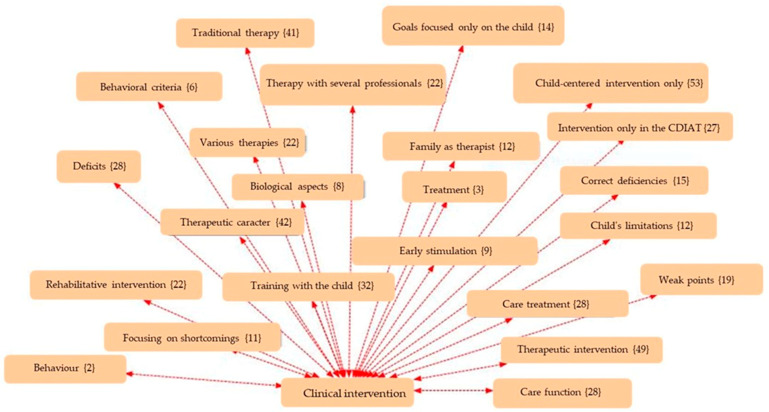
Network of codes associated with the clinical intervention dimension.

**Figure 2:**
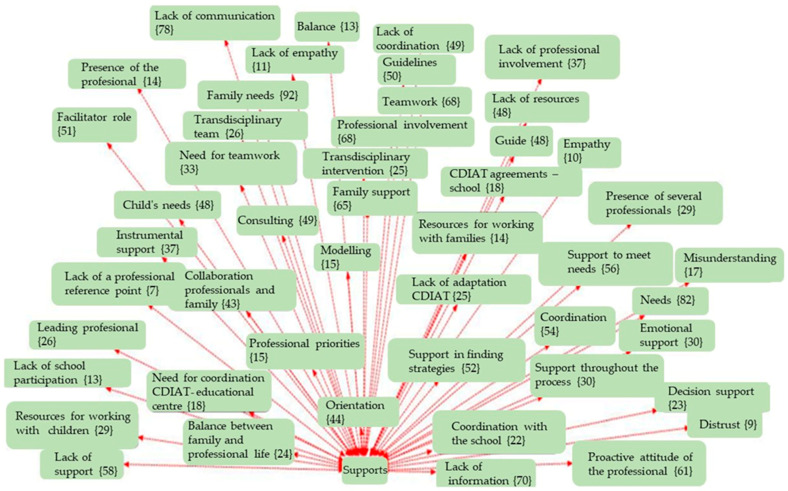
Network of codes associated with the support dimension.

**Figure 3:**
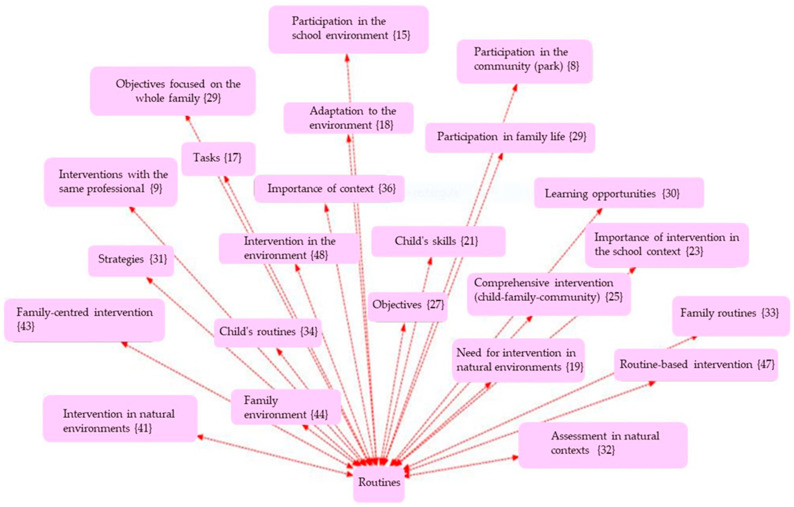
Network of codes associated with the routines dimension.

**Figure 4:**
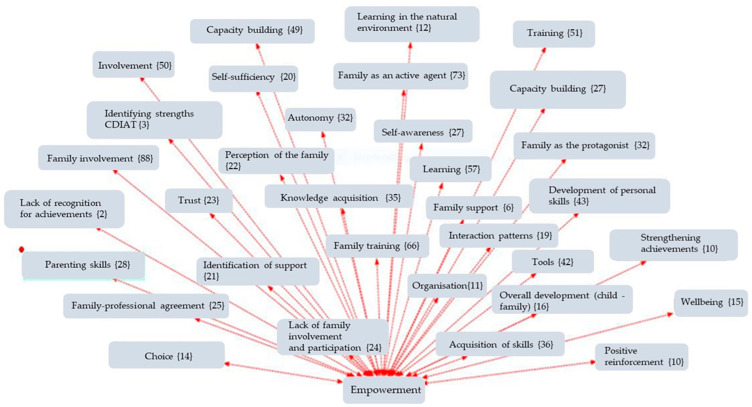
Network of codes associated with the empowerment dimension.

**Figure 5:**
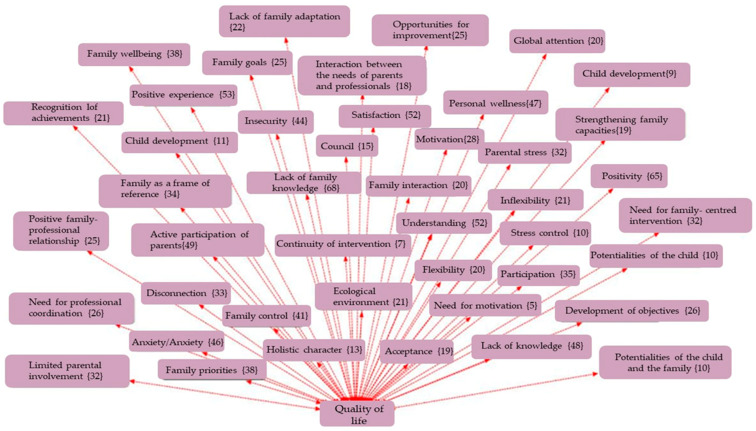
Network of codes associated with the quality of family life.

The analysis was carried out using thematic coding with the support of Atlas.ti (v.9) software, a tool specialised in qualitative analysis of textual data, through which networks of codes and semantic relationships were constructed. An inductive-deductive thematic coding strategy was used, developed in three phases:

- Open coding phase: Where the different units of meaning were identified from the discourses.
- Axial phase: In which the emerging codes were grouped into five main Analytical Dimensions:
  ○ Clinical Intervention (Figure 1);
  ○ Supports (Figure 2);
  ○ Routines (Figure 3);
  ○ Empowerment (Figure 4);
  ○ Quality of Life (Figure 5);
- Finally, the different figures show, in parentheses, the number of times this code was repeated in the responses of the families participating in the 30 interviews.

- Selective phase in which networks of codes and relationships between categories were constructed, allowing for the interpretation of the underlying structures of family discourse.
- These dimensions structured the analysis, allowing us to detect discourse patterns, divergences between the theoretical model and lived experience, and key elements for interpreting the data. The code networks were represented graphically to illustrate the interrelationships between categories, contributing to the visual interpretation of the analysis.
- To validate the category system, a group of experts in early intervention participated, classifying the units of analysis according to their relevance and consistency. The inter-rater agreement index calculated using the Kappa coefficient was 0.83, indicating a high level of reliability in the coding.

## 3. Results

This section presents the findings of the qualitative analysis of the families’ discourses, organised around five emerging thematic dimensions. The categories were identified through an open, axial, and selective coding process using Atlas.ti software. The results are accompanied by representative quotes and visualisations in the form of semantic networks, which illustrate the relationships between the codes grouped under each thematic axis.

### 3.1. Clinical Model Vx. Family-Centred Model

One of the main tensions detected in the families’ accounts was the persistence of a clinical model, focused exclusively on the child, where interventions are carried out in a directive and decontextualised manner. Families describe experiences in which professionals set goals without consulting or actively involving primary caregivers.

This approach contrasts with the routine-based, family-centred model proposed as a theoretical reference, which promotes active participation and shared decision-making. The gap between the two models is cited as a source of frustration by some of the families interviewed.

### 3.2. Professional Support and Communication

Interpersonal relationships with professionals were described as decisive in the care experience. Families value active listening, empathy, and continuity of support, but they complain about the lack of coordination between professionals, frequent changes in therapists, and one-way communication. In this sense, Table 2 presents some of the criticisms expressed by some of the families of the traditional model, as well as some of the virtues of the family-centred model.

There is a significant difference between experiences where professionals adopt a friendly and collaborative attitude and those marked by a hierarchical and distant relationship. In many cases, professionals are perceived as unapproachable and focused on technical tasks (Table 3).

### 3.3. Routines and Natural Contexts

Another notable finding was the lack of contextualisation of the intervention in the child’s everyday environment (Table 4). Most families indicated that the sessions took place exclusively at the centre, with no connection to the usual spaces of family life (home, school, and community).

This lack of adaptation to family routines prevents the proposed strategies from being functional and sustainable. Families ask for flexibility and personalisation, as well as practical training in their own contexts.

### 3.4. Empowerment and Participation

Empowerment was a cross-cutting category that nevertheless appeared unevenly in the narratives. Some families reported having been supported in gaining autonomy and confidence, while others felt displaced by the process (Table 5).

The active participation of families in planning and evaluating objectives remains an unresolved issue. The lack of specific training for professionals in participatory models and institutional rigidity hinder the exercise of shared responsibility and the active involvement of families as essential agents in the intervention to contribute to the overall development of their children.

### 3.5. Quality of Family Life

The final dimension relates to the overall perception of the impact of the intervention on family life. Families who have experienced tailored, personalised, and respectful interventions report improvements in domestic organisation, emotional management, and a sense of control (Table 6).

In this sense, interventions that focus solely on clinical aspects, without considering the overall well-being of the family environment, generate discomfort, emotional overload, and a disconnect between the programme’s objectives and the real priorities of families.

## 4. Discussion

The results obtained reveal a high degree of variability in the actual implementation of Family-Centred Practices (FCP), reflecting the coexistence of two models: on the one hand, a still dominant orientation towards a traditional clinical approach, centred on the professional and artificial contexts; and, on the other, the emergence of isolated practices more consistent with the principles of the model based on routines and natural environments [2,6,28,29,30].

In this regard, the findings of this study coincide with previous research that has highlighted the disconnect between the theoretical model of early intervention and its practical application in public services, marked by limited institutional flexibility, poor professional training in the family-centred model and weak family participation [4,11,13]. Most of the participating families report having experienced intervention processes with little personalisation, one-way communication, and a lack of shared responsibility in decision-making, aspects that limit their empowerment and well-being.

As Dunst et al. [3] have pointed out, the quality of the intervention depends largely on the level of empowerment of families and their perception of competence. In the present study, it was found that interventions carried out in the natural environment, with individualised planning adapted to routines, generate greater security, autonomy, and family participation, favouring a positive emotional climate and greater well-being in the family environment and in their quality of life [8,9].

In contrast, experiences rooted in the traditional clinical model, focused on deficits and the figure of the expert, reproduce hierarchical dynamics, limit family involvement, and place an additional emotional burden on primary caregivers. As Fernández Valero et al. [14] have shown, the absence of contextualisation in the intervention increases the perception of frustration and overload, even affecting the couple’s relationship and the organisation of family life.

### 4.1. Comparison of Models Based on Family Perceptions

Based on the exhaustive analysis carried out, a comparison is made between the Clinical Model and the Family-Centred Model (FCM), using the perceptions gathered in the interviews as the backbone. This comparison allows us to observe the existing gaps and opportunities for improvement from a situated perspective (Table 7).

These results reinforce the findings of recent studies in Spain and Latin America, which warn of the urgent need to strengthen professional training in Family-Centred Practices (FCCP) and promote a more coherent implementation of the principles of the model. To this end, it is essential to redesign services from a logic of co-responsibility, sustainability and empathy [2,10,11].

In this sense, current research has highlighted that the barriers to the real application of the model do not derive exclusively from the individual beliefs of the professional but are strongly conditioned by the organisational structure of the care system, especially in rigid institutional contexts in which the family plays a passive role and does not participate in the intervention [35].

#### 4.1.1. Role and Participation of the Family

Most of the interviews showed a passive participation of families, with unilateral interventions and little opportunity for real involvement. Relevant subcategories as follows: observation without empowerment, feeling of invisibility, and lack of co-responsibility in defining objectives. In the few cases that experienced PCF, families mention that it is part of the choice process, and they are responsible for the implementation of objectives to be worked on, reflecting an active co-responsibility. Previous studies also underline that family involvement is a key predictor of quality in ECI [3,8]. Along these lines, programmes such as SPEEDI [28], COPCA [29], and GAME [30] have shown how training, intervention and family support improve outcomes in newborns with special needs. These studies show how the joint work between specialists (physiotherapists and occupational therapists) and families enhances the future development of newborns in cognitive, linguistic and motor areas.

#### 4.1.2. Professional-Family Communication

Communication is one of the most powerful differentiating factors between the two models. In the traditional clinical approach, technical, hierarchical, and unidirectional communication predominates, with little active listening on the part of professionals. Whereas the family-centred model favours a more collaborative interaction in which parents emphasise openness, empathy, empowerment, and emotional accompaniment. In this sense, the quality of the professional-family bond is revealed as a determining factor in generating trust and making the family feel part of the process, thus favouring the perception of co-responsibility in the intervention process [8,9].

However, this relational dimension is usually limited by the structural conditions of the system, that is, by the very tradition of early intervention practices, by the training of professionals-mostly in the clinical model- and by the very structure of early intervention centres, conditioned to receive the subjects and work with them in their facilities. In addition, the need of certain professionals not to leave their comfort zone also has an influence. The disconnection between the ideals of the model and its actual application has been linked to factors such as the lack of specific technical training in PCF, the absence of protocols that allow for flexible and personalised intervention, and the pre-dominance of a directive clinical framework that inhibits the active participation of families and prevents them from assuming an active role [36].

#### 4.1.3. Context and Intervention Approach

The interviews reveal that the clinical model tends to relegate the intervention to watertight spaces, developed in specialised clinical wards, which generates a disconnection with the infant’s real routines. In contrast, families who experienced interventions aligned with Family-Centred Practices highlight and value the functionality of accompaniment in their own homes or in the infant’s everyday environments, which allowed them to understand aspects of development that were not visible in the clinical context. These findings coincide with international evidence highlighting the need for interventions in natural environments to facilitate meaningful learning adapted to daily life [9,14].

In this line, studies such as the one recently developed in a rural county in Ireland show that contextualised home-based intervention allows observing and working on real dynamics of the family environment, promoting a collaborative and empowering approach, favouring positive results in child development, parental competence, and family quality of life. These experiences indicate that, when the intervention is contextualised and adapted to the real conditions of the family, it promotes greater involvement of caregivers and greater sustainability of the intervention process [37]. In a complementary way, Dias and Cadime [38] found in the Portuguese context that interventions in natural settings strengthen the relationship between professionals and families, facilitate the transfer of functional skills to everyday life, and promote active and empowered participation by caregivers. Also, the GAME programme [30], in intervention with infants at high risk for cerebral palsy, has shown that involving families in developmental activities to enrich the home learning environment yields promising results.

#### 4.1.4. Planning and Adequacy

Another key element identified is the level of personalisation in the planning of the intervention. Families who participated in clinical models reported experiences marked by standardised interventions, poorly adapted to their daily reality, which generated a sense of family imbalance and low personal involvement. On the other hand, those who accessed interventions based on Family-Centred Practices positively valued the practical training received, the application of functional strategies adjusted to their routines, and the co-responsibility in the establishment of objectives. These elements contributed significantly to their empowerment, promoting greater autonomy and quality of family life. This perception coincides with previous studies that support the efficacy of the routine-based approach to foster the active involvement of families and their capacity for action to promote their autonomy [8,11].

In addition, recent research shows the real capacity of families to be actively involved in intervention planning, in this case, using digital tools such as PEM+ (Participation and Environment Measure Plus), an e-health tool for family-centred care. This digital tool allows for improved communication between families and professionals through the determination of priority activities, the establishment of specific objectives, and the identification of intervention strategies in a joint, consensual, and previously known way. The results of the study demonstrate a high proportion of caregivers able to generate participatory care plans aligned with their family reality. This reinforces the idea that personalisation is not only desirable but feasible when adequate resources are provided, allowing the consolidation of family co-responsibility in the definition of goals and strategies to favour their empowerment [39].

#### 4.1.5. Emotional Impact and Family Well-Being

In relation to emotional management, the results reveal that families who participated in clinical intervention models frequently report feelings of overload, frustration, and institutional abandonment. These negative emotional effects are intensified when there is no affective accompaniment or adaptation to the realities of the family environment. In contrast, the family-centred approach favours a more positive experience of the process, promoting emotional support, improved family bonding, and a perception of normality and well-being in everyday life. This significant difference is consistent with the literature linking Family-Centred Practices (FCP) with improvements in family dynamics, emotional adjustment, and psychological well-being of caregivers [14,15].

Several studies, including that of Calero et al. [40], have shown that the use of positive coping strategies by parents, such as cognitive restructuring or active acceptance, is associated with lower levels of parental stress in early care settings [40,41]. However, these strategies are less effective when public services fail to consistently implement PCF, either due to structural barriers, poor professional training, or institutional rigidity, which has a direct impact on family emotional well-being [40,42]. In this sense, there are studies that underline that the authentic application of family-centred practices based on empathic collaboration and co-responsibility has a protective effect against parental stress by reinforcing caregivers’ confidence in their own abilities [28,29,30,41,42]. This is supported by Jeung et al. [43], who show that group-based family resilience programmes, when integrated into community settings, improve parental coping, reduce anxiety, and strengthen parenting skills.

Furthermore, several studies agree that the emotional well-being of families is enhanced when services actively recognise their experience and promote their participation from a respectful and collaborative approach [44]. In this line, the study developed by Della and Cañadas [45], focused on an early care service in Spain, shows that when families are listened to, feel involved in the process, and perceive progress in child development and family quality of life, a positive effect is generated at both an emotional and functional level. The results show how active participation, fluid communication, and access to practical day-to-day strategies contribute to reinforcing the perception of parental competence and empowerment; thus, consolidating more effective and humanised interventions.

In summary, the evidence referred to underlines that the positive emotional impact associated with Family-Centred Practices does not derive exclusively from their conceptual formulation but from their degree of effective, contextualised and sustained implementation over time. The quality of this implementation, mediated by structural, organisational and relational factors, is decisive in promoting empowerment processes, reducing parental stress, and fostering family well-being. Therefore, nominal adherence to the model is not enough; it requires a coherent praxis that articulates specialised professional training, flexible protocols, and a relational approach that actively recognises families as co-responsible agents in the intervention process.

### 4.2. Methodological Contribution: Cross-Coding Matrix

To contribute to a greater visualisation of the interpretative complexity, the following thematic cross-coding matrix is incorporated, which synthesises the emerging categories, the associated codes and the main findings according to the model in which they are framed (Table 8).

## 5. Conclusions

### 5.1. Significance and Implications of the Study

The main objective of the article was to analyse the degree of real implementation of PCF in the Spanish context, from the perspective of the families themselves, identifying points of disconnection between the theoretical model and its practical application, as well as possible areas for improvement and professional adjustment. Ultimately, the aim was also to contribute to improving the quality and coherence of ECI services, promoting more humanised, contextualised and effective practices.

The analysis of family discourses confirms the persistence of a dominant clinical model in many contexts, characterised by a de-contextualised intervention, unidirectional communication, and limited involvement of primary caregivers. However, we also observe the emergence of positive experiences of greater emotional bonding, co-responsibility in decision-making, and family empowerment, all of which are fundamental principles of the routine-based approach and natural environments. These findings reinforce the idea that the paradigm shift requires not only the formal adoption of new methodologies but also a deeper structural and institutional transformation to have a significant impact on improving the quality of life of families.

The results also show the need to improve continuous training in PCF, addressing aspects related to communication skills, joint family-professional planning, and strategies for adaptation to the natural contexts of child and family development. In addition, institutional reforms are needed that prioritise flexible timetables, intervention in the natural environments of the child and family, home visits, the need to establish intervention with a professional reference, and to carry out empowerment evaluation, which implies the design of evaluation tools that consider the effective participation of families as an indicator of quality.

Finally, the results underline the urgency of implementing more humanised, contextualised, and effective practices that improve the quality and coherence of ECI services, such as redesigning early intervention services to favour greater organisational flexibility; training professionals in collaborative and contextualised intervention models; Generating evaluation tools that incorporate the voice of families as an indicator of service quality; and aligning actual practices with international recommendations on early intervention, especially those based on the Routines Model.

### 5.2. Recommendations for Further Research

In summary, this work invites us to continue researching from participatory approaches that place the subjective experience of families at the centre, so that academic research can contribute to transforming, from the base, the policies and practices of childcare. The active participation of families cannot be understood as an optional element but as the backbone of an effective, ethical, and sustainable intervention. Attending to their needs, making their experiences visible, and co-constructing the care process with them is an essential condition for guaranteeing the integral development of the child in their significant environments and improving the quality of life of the family environment.

About future lines of research, we are committed to the development of longitudinal studies on the sustainable effects of interventions centred on families and on the global development of children and families. To carry out comparative studies between autonomous communities to assess the influence of regulatory factors and regional resources on the practices implemented, as well as to carry out triangulation studies of the professional practices implemented, incorporating the perspectives of professionals and other intervention agents to enrich the analysis. In this sense, it would be interesting to analyse the importance of schools and teachers in implementing routines, since they are also a natural environment for children.

### 5.3. Limitations of the Study

Future research could consider expanding the sample with more families, describing the socioeconomic contexts from which they come, and, in this way, being able to establish parameters regarding parental styles and their influence on the implementation of the different models (clinical model and family-centred model). Another limitation of this study is the absence of empirical data that could confirm the opinions of the families provided in the interviews. Such a complement would make the study more solid.

## Figures and Tables

**Table 1:** Number of interviews by autonomous communities.

Autonomous Communities	Number of Interviews
Andalucía	2
Aragón	1
Asturias	1
Cantabria	1
Cataluña	3
Castilla La Mancha	2
Castilla y León	2
Comunidad Valenciana	6
Extremadura	1
Galicia	1
Islas Baleares	1
Islas Canarias	2
La Rioja	2
Madrid	2
Navarra	1
País Vasco	1
Región de Murcia	1

**Table 2:** Representative quotes from families about the clinical model vs. the family centred model.

Subcategory	Representative Quote	Associated Codes (Atlas.ti)	Interpretative Comment
**Professional-centred intervention**	“They told us what to do, but they never asked us what we needed as a family.” (F03, Valencian Community) “The therapist told us what she would do, but she didn’t ask if it was feasible for us.” (F06, Catalonia) “We simply followed instructions.” (F10, Galicia) “They only explained what they were going to do with him, without taking into account what we thought.” (F06, Catalonia) “The approach was always from their perspective, without adapting to what we experienced at home.” (F09, Andalusia) ‘They always told us what to do with the child, but they didn’t ask us if it suited us. We had no say in the matter.’ (F22, Canary Islands)	Lack of participation; clinical model; one-way professional communication	Lack of active listening and unilateral decisions by professionals. The family experiences an imposed intervention, without adaptation or dialogue with the primary carers.
**Activities not contextualised**	“They always told us: now it’s time for this, now we have to do that, but without taking our routines into account.” (F09, Andalusia) “They asked us to do exercises that didn’t fit in with our daily lives.” (F13, Valencian Community) ‘The schedule they proposed did not fit in with our rhythms.’ (F04, Castile-La Mancha) ‘It was like following the same template for everyone.’ (F11, Extremadura)	Clinical model; failure to adapt to routines; imposed activity	A rigid and generalised approach, without considering the family environment.
**Lack of intervention at home**	“They never came to our home, and for us that would have been essential.” (F11, Extremadura) “They never saw how our son lives or how he behaves at home.” (F05, Castile-La Mancha) “The intervention only took place at the centre; they didn’t know our real context.” (F08, Andalusia) “No one was interested in knowing what our daily life was like.” (F03, Valencian Community)	No intervention in the environment; organisational limitations; centre-focused model	Intervention disconnected from the natural environment.
**Institutional rigidity**	“The intervention has to take place within four walls; there is no other alternative.” (F07, Madrid) “They told us they couldn’t come to our home because of the centre’s rules.” (F10, Galicia) “The system is not adapted to family reality.” (F06, Catalonia)	Institutional barriers; clinical model; lack of flexibility	The regulations act as a brake on family-centred practices.
**Poor communication between family and professionals**	“Communication is so poor that… I may be undoing their work.” (F03, Valencian Community) “They gave us guidelines without explaining why they were important.” (F02, Castile and León) “There was hardly any room for asking questions.” (F12, Valencian Community) ‘There were weeks when we didn’t know what had been done with our child.’ (F05, Castile-La Mancha)	Lack of coordination; poor communication; lack of parental training	It affects the continuity and consistency of the intervention.
**Focus on the** **deficit**	“Everything was negative… no one really cared about us.” (F09, Andalusia) “They always talked about what I couldn’t do, not what I could do.” (F13, Valencian Community)	Focus on disability; invisibility of the family; lack of positive approach	A vision focused on deficiencies, without attention to the emotional dimension.
**Lack of work–life balance and** **flexibility**	“I have to ask for the day off… the grandmother can’t take him…” (F06, Catalonia) “The schedules were fixed, with no option to adapt them.” (F07, Madrid) ‘If we arrived 10 min late, they wouldn’t take care of the child.’ (F02, Castile and León) ‘The time is what it is, and it can’t be changed, even if you have a doctor’s appointment.’ (F10, Galicia)	Inflexibility of service; poor work–life balance; rigid model	Family reality and diversity are not considered.
**Lack of individualised planning**	“We had a mixed model… but it didn’t suit our needs.” (F01, Valencian Community) “The plan they followed was the same for everyone.” (F09, Andalusia)	Standardised intervention; lack of personalised assessment; lack of contextualisation	It does not fit in with family priorities or dynamics.
**Lack of systemic vision**	“They have never been interested in knowing what our family is like or what we do on a daily basis.” (F01, Valencian Community) “They only talked about the child, as if he were alone in the world.” (F15, Aragon)	Lack of contextual knowledge; lack of networking; expert model	Lack of knowledge about family structure is the basis for the process.

**Table 3:** Representative quotes from families and coding associated with professional support and communication.

Subcategory	Representative Quote	Associated Codes (Atlas.ti)	Interpretative Comment
**Lack of active listening**	‘The therapist explained things to us, but she didn’t listen to us. We felt that we couldn’t express what was worrying us.’ (F06, Catalonia) ‘It seemed like we were there to receive instructions, not to participate.’ (F20, Navarre) ‘Everything was prepared in advance, there was no opportunity to speak.’ (F03, Valencian Community)	One-way communication; invisibility of the family; lack of listening	The professional role remains hierarchical and limits dialogue with the family.
**Absence of professional relationship**	“A different person came every time. We never managed to build a relationship of trust.” (F14, Madrid) “The relationship with the therapists was so brief that there was no opportunity to get to know each other.” (F09, Andalusia) “They change so often that you don’t even know the name of the next one.” (F11, Extremadura)	Frequent changes; lack of continuity; professional instability	Staff turnover prevents the building of stable and reliable relationships.
**Hierarchical** **relationship**	‘They tell you what to do, period. They don’t ask how things are going at home.’ (F09, Andalusia) ‘It was like following orders; there was no room for discussion.’ (F15, Aragon) ‘I always felt that the therapist was in charge and we obeyed.’ (F01, Valencian Community)	Professional approach; lack of family involvement; expert model	The family is relegated to a passive position, with no decision-making power.
**Lack of** **coordination**	‘Sometimes they would tell us different things, and that confused us a lot.’ (F12, Valencian Community) ‘Each therapist gave us a different message.’ (F05, Castile-La Mancha) ‘No one knew what the other had done, everything was repeated.’ (F15, Aragon)	Lack of coordination; interprofessional contradictions; communication insecurity	Inconsistency between statements generates uncertainty and mistrust.
**Collaborative communication**	“Our therapist always asked us how we were doing, what was worrying us… and that made us feel part of the process.” (F03, Valencian Community) “From day one, we felt supported, not judged.” (F20, Navarre) “They gave us space to talk, and that helped a lot.” (F18, Region of Murcia)	Active listening; emotional connection; professional support	Openness to dialogue facilitates partnership and family empowerment.
**Assessment of continuous support**	“When we found out that the same therapist was always coming, we breathed a sigh of relief. We already knew how to work with her.” (F05, Castilla-La Mancha) “Continuity allowed us to move forward with confidence.” (F15, Aragón) “We understood each other without speaking, which only comes with time.” (F06, Catalonia)	Professional continuity; stable relationship; climate of trust	Continuity in professional relationships promotes consistent planning.
**Inaccessible professional**	“We didn’t know when he would come, or if we could call him if something happened.” (F11, Extremadura) “During holidays or public holidays, we had no one to contact.” (F09, Andalusia) “We asked by post, and they didn’t reply.” (F05, Castile-La Mancha)	Professional inaccessibility; lack of follow-up; professional disengagement	Poor accessibility limits emotional and technical support during the process.
**Perceived** **emotional** **support**	‘Our psychologist gives me security, support, and lifts my spirits.’ (F06, Catalonia) ‘She wasn’t just concerned about the child, but also about how we were doing.’ (F14, Madrid) ‘She knew when we needed to talk and when we just needed to listen.’ (F13, Valencian Community)	Emotional support; professional trust; empathetic relationship	Support that goes beyond technical matters and addresses emotions is highly valued.
**Poor communication**	“I get my information from others. The information I receive from the sessions is very limited.” (F15, Aragon) “They just told us ‘It’s better’ or ‘it’s worse’, but we didn’t know why.” (F13, Valencian Community) “We never knew what goal they were working towards.” (F20, Navarre)	Lack of information; fragmented communication; parental disengagement	The lack of clarity and transparency limits learning and active participation by families.
**Effective coordination**	“You consult with a therapist who acts as an intermediary […] they always give you an answer.” (F14, Madrid) “The whole team was aware of the situation, and that gave me security.” (F09, Andalusia) “The therapists talked to each other, which meant we didn’t have to repeat things.” (F11, Extremadura)	Professional coordination; shared monitoring; fluent communication	Teamwork improves the consistency and reliability of the process.
**Comprehensive support**	‘The centre organises groups for parents, grandparents, siblings […] it was very enriching.’ (F14, Madrid) ‘Participating in workshops with other families helped us to normalise what was happening to us.’ (F13, Valencian Community) ‘We feel part of a network.’ (F06, Catalonia)	Community support; extended family inclusion; group dynamics	The value of care that includes extended family networks and shared support spaces is recognised.

**Table 4:** Representative quotes and coding associated with routines and natural contexts.

Subcategory	Representative Quote	Associated Codes (Atlas.ti)	Interpretative Comment
**Intervention in artificial contexts**	“The care was always provided at the centre, in a room with toys, but that has nothing to do with our home.” (F08, Andalusia) “Everything took place in a specially prepared space, which had nothing to do with our everyday life.” (F01, Valencian Community)	Decontextualised intervention; clinical model; poor functional transfer	The intervention takes place in an artificial environment, disconnected from the child’s real routines.
**Absence of home intervention**	“It would have helped us if they had come to our home or seen what everyday life is like, but that was never considered.” (F14, Madrid) “No one ever asked to see what our son’s real environment was like.” (F03, Valencian Community) “I would have felt more supported if they had seen how we do things at home.” (F24, Catalonia)	Lack of home visits; lack of personalisation; professional distance	Families miss the support provided in the child’s natural environment.
**Lack of connection to school**	“They never asked us what his routine was like at school, nor did they help us with that.” (F15, Aragon) “School was like another world, nobody coordinated anything.” (F06, Catalonia)	Lack of educational coordination; absence of intervention in the classroom; poor contextualisation	Lack of knowledge about the school context prevents a comprehensive approach to child development.
**Limited customisation of strategies**	“They gave us general advice, but it didn’t fit with our day-to-day reality.” (F25, Castilla-La Mancha) “They recommended things that didn’t fit with how we organise ourselves.” (F10, Galicia)	Lack of adaptation; generic advice; no contextualisation in routine	Strategies that are not highly individualised are difficult to implement in a real family environment.
**Desire for functional intervention**	“We wanted them to teach us how to act at specific times of the day, such as at mealtimes or bath time.” (F06, Catalonia) “What we needed most was to know how to handle real situations, and that was never worked on.” (F13, Valencian Community)	Functional intervention; family routines; need for practical training	Families demand practical guidance tailored to everyday routines.
**Lack of organisational flexibility**	“We could only go to the centre at a specific time, and it didn’t suit our needs.” (F26, Castile and León) “They couldn’t change the time, even if it was for something important to us.” (F25, Castile-La Mancha)	Institutional rigidity; poor work–life balance; lack of accessibility	The rigid structure of the service does not allow for family-centred intervention tailored to their context.
**Need for work in natural environments**	“If they had accompanied us at home or seen how we do things, it would have been more useful for everyone.” (F13, Valencian Community) “Understanding how we live is key, and that cannot be achieved in a white room.” (F15, Aragon)	Work in context; family involvement; professional proximity	Families associate the effectiveness of intervention with direct involvement in their environment.
**Focus on family routine**	“They helped us reorganise our schedules and understand what things we could do more easily at home.” (F03, Valencian Community) “When work adapts to our routines, everything flows better.” (F11, Extremadura)	Functional support; family empowerment; intervention in routine	A positive example of intervention tailored to everyday life that empowers and facilitates autonomy within the family.
**Disruption to family routine**	“They told us to repeat the exercises, but sometimes they didn’t fit in with our schedules or our family dynamics.” (F10, Galicia) “We had to reorganise everything to be able to apply what they told us.” (F25, Castilla-La Mancha)	Unworkable strategies; failure to adjust to family routine; added burden	The intervention is perceived as an additional requirement, not integrated into the family’s daily life.
**Activity-based intervention**	“During the session, they did things with him, but then we didn’t know how to continue at home.” (F25, Castilla-La Mancha) “It was like a show there, but without translation at home.” (F14, Madrid)	Intervention not very sustainable; little transfer to the home; lack of continuity	Families point out the lack of practical tools to continue therapeutic work at home.
**Relevance of the natural environment**	“When he came home, he understood many things that weren’t apparent at the centre. It was very different.” (F11, Extremadura) “He behaved one way there, but at home it was a different world. And that was never apparent.” (F09, Andalusia)	Contextual observation; reality-centred intervention; functional assessment	Intervening in everyday environments allows for a better understanding of real needs.
**Lack of resources for follow-up**	“They sent us some sheets, but we didn’t know if we were doing it right. They never came to check.” (F15, Aragon) “There was a lack of support in applying what they told us.” (F24, Catalonia)	Lack of supervision at home; family insecurity; poor practical training	The family does not feel supported or encouraged to implement career guidance in their context.

**Table 5:** Representative quotes and coding associated with empowerment and participation.

Subcategory	Representative Quote	Associated Codes (Atlas.ti)	Interpretative Comment
**Lack of practical empowerment**	“We wanted them to explain how to do things at home, but they only did it with the child in the room.” (F05, Castilla-La Mancha) “The sessions were only for the child. We watched from outside.” (F18, Murcia Region) “They never taught us how to intervene, we just observed.” (F12, Valencian Community)	Intervention focused on the professional; lack of practical training; passive observation	Families express an intervention focused on the technician, without functional transfer to their active role.
**Doubts and feelings of inadequacy**	“We had many doubts and did not feel equipped to help. We just stood by and watched.” (F13, Valencian Community) “I didn’t know if I was doing it right. I was afraid of making a mistake.” (F02, Castile and León)	Lack of empowerment; low perception of competence; passive role	It reflects the absence of strategies that promote family autonomy.
**Limited participation in decisions**	“They told us what we had to work on, but they never asked us what worried us most.” (F14, Madrid) “They came with everything already decided. No one asked for our opinion.” (F06, Catalonia)	One-way communication; little shared responsibility; lack of listening	Goal planning is performed without consulting family priorities.
**Desire for greater involvement**	“I wanted to learn how to stimulate him myself, but they didn’t explain how. I felt useless.” (F15, Aragon) “We wanted to be more involved, but they didn’t give us a chance.” (F24, Catalonia)	Need for empowerment; professional intervention; frustration	The lack of guidance limits real involvement and generates feelings of insecurity.
**Feeling of invisibility**	“It seems that we are the only ones who bring the child, but no one takes us into account.” (F06, Catalonia) “Sometimes they didn’t even look at us. They just talked among themselves.” (F09, Andalusia)	Passive role of the family; lack of appreciation; expert model	The familiar figure becomes blurred in the process of intervention.
**Example of positive accompaniment**	“They helped us a lot to understand what we could do with her. We felt more relaxed and confident.” (F08, Andalusia) “I finally felt that someone was teaching me and giving me confidence.” (F13, Valencian Community)	Family empowerment; safety; applied training	Reflection of an intervention based on training and active support.
**Real shared responsibility**	“We chose the objectives together, based on what we needed most at home.” (F02, Castile and León) “They always asked us what was important to us.” (F10, Galicia)	Joint planning; active participation; co-decision	Example of a practice aligned with the family-centred model.
**Useful training for independence**	“They taught us how to play with her in a way that was also therapeutic. We learned a lot.” (F04, Castile-La Mancha) “Thanks to what we learned, we now know how to help her on a daily basis.” (F03, Valencian Community)	Functional strategies; practical learning; family training	It shows the impact of empowerment on everyday family life.
**Non-participatory expert model**	“They knew what they were doing, but they didn’t explain anything to us. We didn’t feel part of the process” (F10, Galicia) “They were always very technical, but distant with us” (F01, Valencian Community) “They knew a lot, of course, but they didn’t explain to us what they were doing or why. We just watched from the outside.” (F23, Canary Islands)	Expert model; lack of transparency; disconnection from the family	It illustrates a hierarchical and professional intervention that excludes the family from shared decision-making. The family feels excluded from decisions and the therapeutic process, relegated to a passive role.

**Table 6:** Emerging results from the analysis of the dimension: quality of family life.

Subcategory	Representative Quote	Associated Codes (Atlas.ti)	Interpretative Comment
**Improvement in family dynamics**	“When the intervention is tailored to who we are, not only does the child improve, but we also improve as a family.” (F07, Castilla-La Mancha) “Knowing how to help my son has also improved our relationship as a couple.” (F08, Andalusia) “Before, I didn’t know how to do things, and neither did my partner… my family has improved since we started going to the centre.” (F04, Castilla-La Mancha)	Contextualised intervention; Family adjustment; Positive impact on the family	A respectful and flexible approach has a direct impact on family harmony and balance.
**Positive emotional support**	“A good professional makes all the difference; she helped us feel more capable and less overwhelmed.” (F01, Madrid) “Our psychologist gives me security, support, lifts my spirits…” (F02, Madrid) “She always motivates us… that has a big impact on whether you go home feeling cheerful or not.” (F06, Catalonia)	Emotional support; professionalism; stress reduction; emotional bond with professionals	It highlights the protective effect of quality professional relationships on family emotional management.
**Emotional overload**	“Every appointment was stressful. We didn’t really know why we were going, or if it was useful.” (F03, Andalusia) “We just went through the motions, but it was often more exhausting than helpful.” (F12, Valencian Community)	Family stress; insecurity; emotional overload; lack of guidance	A lack of clarity and realistic objectives in the intervention can lead to exhaustion and demotivation.
**Negative impact of the clinical model**	“It was like taking him to the doctor. It was all about measuring and testing, but nothing changed at home.” (F09, Catalonia) “Everything is centred… we don’t have a purely family-centred model.” (F05, Andalusia)	Clinic intervention; limited functionality; family disengagement	Describe an experience in which professional intervention does not translate into real benefits for the family.
**Feeling of institutional abandonment**	“When we needed it most, they stopped our therapy because they said we no longer met the requirements.” (F06, Catalonia) “They cut it off suddenly, as if it were no longer necessary. We felt very alone.” (F14, Castile and León) “One day they told us there would be no more sessions because it was no longer necessary. It was sudden, without explanation. We felt abandoned.” (F22, Canary Islands)	Lack of continuity; institutional rigidity; frustration	The system creates vulnerability when decisions do not consider the emotional process of the family. The family experiences an abrupt end to intervention, without support or emotional containment.
**Recognition of positive changes**	“Since we started working together, I see my daughter happier, and I also feel more confident as a mother.” (F08, Andalusia) “It helps us and is great for us.” (F02, Madrid)	Family empowerment; emotional well-being; functional intervention	It expresses how the collaborative approach improves not only the child, but also the family’s self-esteem and confidence.
**Disconnect between objectives and reality**	“Sometimes they suggested things that didn’t make sense to us. We were overwhelmed, and they talked about stimulating us even more.” (F11, Galicia) “They suggested activities that didn’t fit in with what we were experiencing at home. It frustrated us even more.” (F03, Andalusia)	Mismatch of expectations; overload; lack of listening	It shows a lack of alignment between the professional proposal and the real needs of the family.
**Influencia en la percepción de normalidad**	“Thanks to how they approached it, we felt that we weren’t so different. They helped us see what we could do” (F02, Castile and León) “More than our daughter’s needs, it’s the family’s needs… it’s the family that they prioritise there” (F09, Valencian Community) “In the family group, we saw that we weren’t alone, that other people were going through similar things. That helped us see everything more calmly.” (F21, Balearic Islands)	Family inclusion; positive perception; emotional reinforcement; comprehensive approach	It highlights an intervention that promotes acceptance, self-esteem, and a sense of family normality. Participation in group spaces reinforces the sense of belonging and reduces anxiety about difference.

**Table 7:** Comparison of intervention models from the family’s perspective.

Key Dimension	Clinical Model (Families’ Perception)	Family-Centred Model (Families’ Perception)
**Role of the family**	Passive, limited to observation or compliance with guidelines.	Active, with decision-making, participation and learning skills.
**Intervention context**	Specialised centres, artificial rooms, little connection with the real environment.	Address, community, daily routine of the child.
**Professional-family communication**	Unidirectional, technical, with little listening and emotional support.	Collaborative, relationship-based, active listening and support.
**Goal planning**	Standard, without consulting the family, focused on areas of development.	Functional, defined together with the family, based on everyday priorities.
**Family empowerment**	Low, with feelings of inadequacy, insecurity, and invisibility.	High, promoting confidence, autonomy, and capacity for action.
**Emotional perception of the process**	Frustration, emotional overload, institutional stress.	Well-being, motivation, positive relationship with professionals.

**Table 8:** Cross coding matrix.

Dimension	Specific Subcategories	Code Network Associated (Atlas.ti))	Model Dominant Identified	Correspondence Theoretical (Clinical vs. PCF)
**1. Family role and participation**	Passive observation; Lack of practical empowerment; Feeling of invisibility; True co-responsibility	Passive role, lack of training, invisibilisation, joint planning	Mostly clinical, with isolated cases of PCF	Clinician → Passivity/PCF → Activation and co-responsibility
**2. Professional communication family**	One-way communication; Lack of active listening; Collaborative communication; Emotional support	Technical communication, poor listening, emotional support, professional bonding	Mixed, clinical tendency	Clinical → Instruction/PCF → Dialogue, linkage
**3. Context and intervention approach**	Intervention in artificial spaces; Absence of home visits; Little action in school; Work in the home	Artificial centre, lack of visitors, school disengagement, interference in routines	Dominant clinician, emerging PCF	Clinical → Centres/PCF → Natural environments
**4. Planning and adaptation**	Lack of personalisation; Misalignment of goals; Empowerment training	Standard strategies, clinical objectives, practical empowerment	Clinical predominance with PCF cores	Clinical → Standard/PCF → Individualised and functional
**5. Emotional impact and well-being**	Emotional overload; Feeling of institutional neglect; Family-family improvement; Perception of normality	Stress, lack of monitoring, emotional support, positive perceptions	Dual: strong contrasts between the two models	Clinical → Frustration/PCF → Well-being and acceptance

## Data Availability

The original contributions presented in this study are included in the article. Further inquiries can be directed to the corresponding author.
